# Genetic Mouse Models to Study Pancreatic Cancer-Induced Pain and Reduction in Well-Being

**DOI:** 10.3390/cells11172634

**Published:** 2022-08-24

**Authors:** Michael Hirth, Yong Xie, Christiane Höper, Amandine Prats, Thilo Hackert, Matthias P. Ebert, Rohini Kuner

**Affiliations:** 1 Pharmacology Institute, Medical Faculty Heidelberg, Heidelberg University, 69120 Heidelberg, Germany; 2 Department of Medicine II, Medical Faculty Mannheim, University of Heidelberg, 68167 Mannheim, Germany; 3 European Molecular Biology Laboratory, 69117 Heidelberg, Germany; 4 Department of General, Visceral and Transplantation Surgery, Heidelberg University Hospital, 69120 Heidelberg, Germany

**Keywords:** pancreatic ductal adenocarcinoma, pain, nerve hypertrophy, KPC, KPPC, cytokines

## Abstract

In addition to the poor prognosis, excruciating abdominal pain is a major challenge in pancreatic cancer. Neurotropism appears to be the underlying mechanism leading to neuronal invasion. However, there is a lack of animal models suitable for translationally bridging in vitro findings with clinical trials. We characterized KPC (Kras^G12D/+^; Trp53^R172H/+^; P48-Cre) and KPPC (Kras^G12D/+^; Trp53^R172H/R172H^; P48-Cre) mice with genetically determined pancreatic ductal adenocarcinoma (PDAC) and compared them with an orthotopic pancreatic cancer mouse model, healthy littermates and human tissue. We analyzed behavioral correlates of cancer-associated pain and well-being, and studied neuronal remodeling and cytokine expression. Histologically, we found similarities between KPC and KPPC tissue with human samples. Compared to healthy littermates, we detect nerve fiber hypertrophy, which was not restricted to a certain fiber type. Interestingly, while KPPC mice showed significantly reduced well-being, KPC mice emerged to be better suited for studying long-lasting cancer pain that emerges over a slow course of tumor progression. To address the neuroinflammatory correlate of loss of well-being, we studied cytokine levels in KPPC mice and observed a significant upregulation of CXCL16, TNFRSF5, CCL24, CXCL1, CCL22, CLL20 and CX2CL1. In summary, we demonstrate that the KPC mouse model is best suited to studying cancer pain, whereas the KPPC model can be employed to study cancer-associated reduction in well-being.

## 1. Introduction

Worldwide, pancreatic ductal adenocarcinoma (PDAC) is diagnosed in over 450,000 people per year [1]. PDAC is one of the most malignant tumor entities, as demonstrated by the low 5-year survival rate of approximately 6% [2]. The low 5-year survival rate is caused by the aggressive tumor biology, and particularly by the absence of early symptoms. Therefore, many patients are already in advanced tumor stages at the time point of diagnosis [2]. Whereas only 30–40% of patients report abdominal pain at the time of diagnosis, up to 80% of patients develop tumor pain as the disease progresses [3]. Every second patient described cancer pain to be severe. The description of PDAC-associated pain can differ. Most patients report abdominal or back pain [3]. However, in some patients, pain can also occur secondarily, e.g., in case of nerve impingement, duodenal stenosis or metastasis [4,5,6]. When the disease progresses, many patients report chronic excruciating abdominal pain [3]. Thus, adequate treatment of cancer pain is an important goal in order to maintain quality of life [3]. The pathophysiology of cancer pain is complex and multifactorial [3,4]. However, it seems that neuronal invasion of cancer cells plays an important role, leading to alterations in the neuronal compartment [7,8,9,10]. Especially in patients suffering from severe pain, pronounced neuronal remodeling can be observed, which is characterized by nerve fiber hypertrophy and increased nerve fiber density [8]. This might be the reason why our current therapeutic strategies are inefficient. Instead of a symptomatic treatment with opioid and non-opioid analgesics, we need a mechanistic treatment approach.

Due to its frequent occurrence and massive impairment of patients’ quality of life, research into molecular and cellular mechanisms of cancer pain are a priority. In order to render this feasible, models for the study of cancer pain and changes in quality of life are particularly necessary. Since the percept of pain and well-being cannot be modeled in cell cultures, animal models are particularly necessary. In recent years, various pancreatic tumor models have been established, which include either the extracorporeal introduction of tumor cells or a genetically modified animal model. These animal models have significantly improved our understanding of tumor biology, but it is still unclear, in the case of almost all PDAC animal models, whether they model the pronounced neuronal reorganization and tumor pain seen in humans. Recently, for the first time, we examined a mouse model after orthotopic injection of PDAC cells into the pancreas to report robust tumor pain [11]. However, genetically engineered mouse models have not yet been validated for their usability to study PDAC-associated pain. The most frequently used genetically modified mouse models harbor mutations in Kras and p53 [12]. Accordingly, activating mutations in KRAS are found in up to 90% of human specimens [13]. Additionally, inactivating mutations are found most frequently in tumor suppressor genes, such as p53 and CDKN2A [13]. Pancreas-specific Cre (e.g., P48-Cre) are commonly crossed with Kras^G12D^ and mutated tumor suppressors (e.g., Trp53^R172H^). The KPC mouse model (Kras^G12D/+^; Trp53^R172H/+^; P48-Cre) is the most frequently used engineered mouse model in pancreatic cancer research [14,15]. Delahoussaye et al. found that the same chemotherapies used in humans are effective in KPC animals [16]. The KPC model is furthermore used to test innovative therapeutic strategies such as immunotherapy and anticancer vaccination [17,18]. The KPPC mouse model harbors homozygote p53 mutation and is also used in the field. For example, Strand et al. demonstrated that siRNA reduced the KRAS-driven cancer growth [19].

In our current work, we characterized the KPC (Kras^G12D/+^; Trp53^R172H/+^; P48-Cre) and KPPC mouse models (Kras^G12D/+^; Trp53^R172H/R172H^; P48-Cre), which represent heterozygous and homozygous mutations in p53, with regard to cancer pain, reduction in well-being, neuronal remodeling and cytokine expression profile in the tumor tissue.

## 2. Materials and Methods

### 2.1. Mouse Strains and Testing Procedure

Kras^G12D/+^; Trp53^R172H/+^; P48-Cre (KPC) and Kras^G12D/+^; Trp53^R172H/R172H^; P48-Cre (KPPC) mice were generated by crossing Lox Stop Lox (LSL) Kras^G12D/+^ and LSL Trp53^R172H/R172H^ animals with P48-Cre animals. Mice from the same mating lacking P48-Cre were used as controls to prevent bias due to changes in the environmental condition.

**Figure 1:**
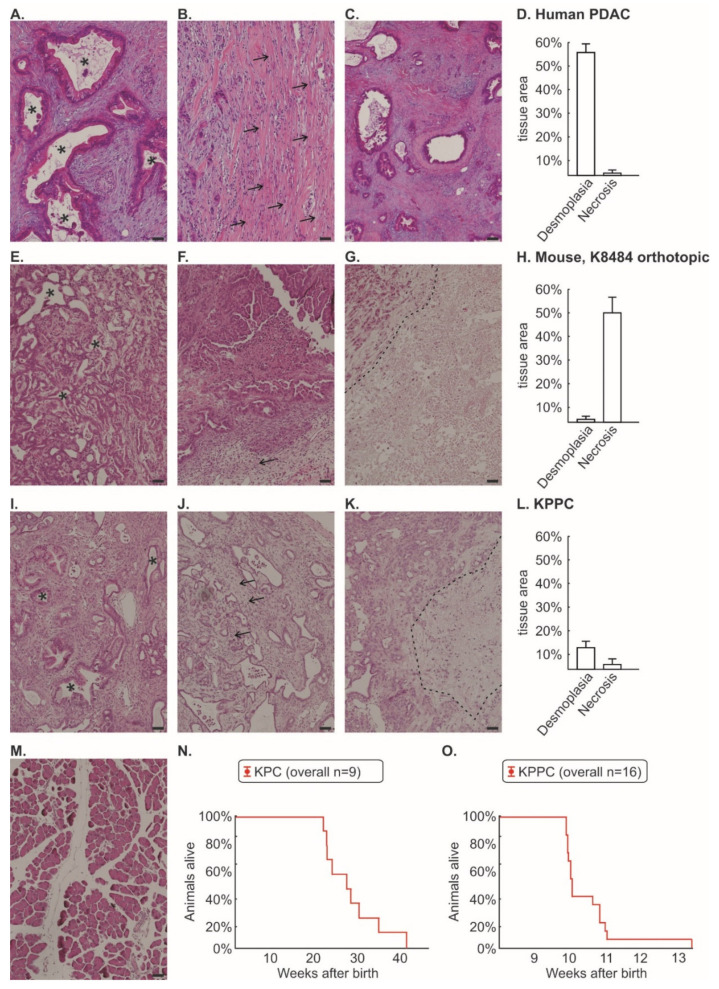
**Histological comparison between human pancreatic cancer, murine orthotopic K8484 injection, KPC mice and KPPC mice.** Post mortem histological staining of pancreatic tissue was performed using Hematoxylin and Eosin (H&E) in human pancreatic cancer (n = 24, (**A**–**C**)); murine cancer model with orthotopically injected K8484 cells (n = 13; (**E**–**G**)); KPPC mice (n = 11; (**I**–**K**)) and control mice (**M**). The areas of desmoplasia and necrosis were quantified for human pancreatic cancer (**D**), murine cancer model with orthotopically injected K8484 cells (**H**) and KPPC mice (**L**). Representative images are shown for the typical duct-like formation of PDAC cells (**A**,**E**,**I**), desmoplasia (**B**,**F**,**J**) and necrosis (**C**,**G**,**K**). Scale bar = 100 µm. Asterisks mark duct-like structures which are typical for PDAC. Arrows mark desmoplasia. The necrotic area is separated by a dotted line from vital pancreatic cancer tissue. (**N**,**O**). Kaplan–Meier curves of 9 KPC mice and 16 KPPC mice were shown in (**N**,**O**).

After genotyping, mice were distributed to cages with 4–5 animals per cage. Each cage contained KPC or KPPC and control mice. Animals were acclimatized for one week. At the age of 15 weeks, KPC mice were scheduled for experiments. The experiments were terminated 27 weeks after birth. In contrast, KPPC mice show significant fast tumor progression, as described by Bardeesy et al. [20] (also see Figure 1N). Thus, KPPC mice were scheduled for experiments at 8–10 weeks, owing to which the n numbers in the experimental setting decreased quickly. As soon as there were no more KPPC mice in a cage, the remaining control mice from this cage were not used for further experiments.

All procedures were in accordance with local ethical guidelines and were approved by the local governing authority (Regierungspraesidium Karlsruhe, Germany; approval numbers: 35-9185I.81/G-126/16; 35-9185.81/G-233/14).

### 2.2. Orthotopic Mouse Model

The orthotopic mouse model of pancreatic cancer involving K8484 cells injection was employed as described previously [11]. The K8484 cell line was established from KPC transgenic mice [12]. Briefly, 50,000 K8484 cells in Matrigel/PBS were injected into the head of the pancreas under microscopic visual control in anesthetized C57BL/6 mice.

### 2.3. Pain Analysis in Mice

Animals were housed with free access to food and water under the standard 12 h light/dark cycle.

Behavioral analyses were performed as described previously [11]. To test cancer-induced abdominal mechanical hypersensitivity, we applied increasing punctuate pressure with von Frey filaments to the abdomen of the mice, applying each filament five times. The frequency of nocifensive responses was noted, and the mechanical threshold was calculated as the force evoking at least 40% frequency of nocifensive responses to abdominal stimulation.

Data were presented as a cumulative integral response, which was calculated as an integral of a stimulus–response function for filaments 0.008 g–0.16 g.

### 2.4. Behavioral Analysis of Well-Being

To evaluate the well-being of mice, we used the open-field test to analyze the exploratory behavior of mice. Mice were introduced individually in the open-field chamber and video graphed for 10 min. Several behavioral parameters (e.g., time immobile, speed) were analyzed automatically. The open field test is a well-known test for behavioral analysis, which has been used for several decades [21,22]. Even though the test is not absolutely specific, it is a good tool to quantify well-being [23,24]. In former analyses, we could already demonstrate the usability of the test to evaluate well-being in PDAC [11,25].

Additionally, we employed Laboras home-cage observation system (Metris B.V.), which detects behavior-specific vibration patterns of the mice over time and processes them into various behavioral parameters (e.g., climbing, locomotion). Home cage monitoring was performed over 24 h and was repeated once per week. It is well documented that pharmacological as well as non-pharmacological interventions which have an impact on well-being can be quantified with the LABORAS test [26,27].

Furthermore, mice were placed individually in cages containing a running wheel with free access to food and water. Voluntary wheel running activity was recorded over 24 h.

### 2.5. Histopathology and Immunohistochemistry

At the end of the behavioral experiments, mice were perfused with 4% PFA, and pancreatic tissues including the cancer were dissected out. Sections were cryoprotected with 30% sucrose and cut using a cryotome (CM3050S; Leica, Wetzlar, Germany). Pancreatic tissue samples from PDAC patients who had undergone resection were provided by the EPZ-Pancobank at the Department of Surgery (approval number: 301/2001, S-708/2019), University Clinic Heidelberg, Germany, in accordance with the regulations of the tissue bank and the approval of the ethics committee of Heidelberg University. Human tissue was provided as paraffin sections.

For immunohistochemical analysis, paraffin sections were re-hydrated and antigen retrieval was performed with sodium citrate, as previously described in detail [11]. After blocking for 60 min with 10% normal horse serum, primary antibodies were applied overnight at 4 °C. We used the following primary antibodies: Anti-β-tubulin III (1:500, #T2200, Sigma Aldrich, St. Louis, MO, USA) and PGP 9.5 (1:500, #PA1-46204, ThermoFisher Scientifics, Waltham, MA, USA) as phenotypic marker for peripheral neurons, Anti-Calcitonin Gene Related Peptide (Anti-CGRP; 1:300; #24112; ImmunoStar, Herford, Germany) as marker for peptidergic nociceptive fibers, Anti-NF200 (1:700; #CH23015; Neuromics, Edina, MA, USA) as marker for A beta fibers and Anti-Tyrosine Hydroxylase (Anti-TH; 1:300; #ab76442; Abcam, Cambridge, UK) as marker for sympathetic fibers. Anti-panCK (1:500, #ab9377; Abcam) was used to identify pancreatic cancer cells. Subsequently, sections were incubated with appropriate secondary Alexa-conjugated antibodies (1:500; Dianova, Hamburg, Germany) for one hour at room temperature.

For Hematoxilin & Eosin (H&E) staining, paraffin sections were re-hydrated, dipped in 1% acid water, stained in hematoxylin for 7 min, washed with water for 3 min, stained in Eosin for 15 s and then dipped in 1% acid water.

We quantified the nerve diameter using ImageJ, as described previously [28]. Depending on the cancer volume, several sections per mouse (median: n = 6 per mouse) covering the whole pancreas were analyzed. The mean nerve diameter and number of nerves/cm^2^ per animal was used for further analysis.

### 2.6. Protein Array

Using the Cytokine Array #AAM-CYT-2000 (RayBio^®^ C-Series Mouse Cytokine Antibody Array C2000), we were able to detect 144 different cytokines in pancreatic tissue. We perfused the mice with PBS post mortem and removed the pancreatic tissue including the tumor. Mechanical lysis was performed prior to normalizing to the total amount of protein. The array was then tested according to the manufacturer’s instructions. The membranes were scanned and the signal intensity was quantified using ImageJ. Membranes with an irregular background were excluded from the analysis. The signal intensity of each dot was normalized to the positive control (=100). Duplicates of each cytokine were analyzed in eight KPPC mice and in six control mice (“control”).

### 2.7. Data Analysis and Statistics

We performed all statistical tests using Statistica, release 7.1 (StatSoft, Tulsa, OK, USA). Unless stated otherwise, data were presented as mean ± S.E.M. and one-way ANOVA of random measures or ANOVA of repeated measures was implemented. Student’s *t*-test was performed for comparisons between two metric parameters. *p* < 0.05 was considered statistically significant. All reported *p* values were two-sided.

## 3. Results

Genetically engineered mouse models carrying mutations in p53 and KRAS are frequently used to study pancreatic cancer. In this study, we investigated whether the KPC and KPPC mouse models are suitable for translational research on cancer pain, cancer-induced reduction in well-being and neuronal remodeling in PDAC.

### 3.1. Tissue from KPC and KPPC Animals Resembles Human Tissue Better Than an Orthotopic Mouse Model of Injected K8484 Cells

We first performed a histological comparison between human PDAC tissue (Figure 1A–D), orthotopically injected K8484 cells into healthy C57BL/6 mice (Figure 1E–H), tissue from KPPC animals (Figure 1I–L) and control mice (Figure 1M). It is worth mentioning that K8484 cells were originally isolated from KPC tumors (Kras^G12D/+^; Trp53^R172H/+^; P48-Cre) [12]. However, the orthotopic injection of K8484 cells, which develops a very robust pain phenotype [11,28], showed a significant loss of desmoplastic tissue areas and a substantial proportion of necrotic areas compared to human tissue (Figure 1D,H). In particular, the large proportion of necrotic area appears artificial. We could not identify relevant histological differences between KPC and KPPC tissue (quantification given for KPPC in Figure 1I–K). Tissue from KPPC animals showed less fibrotic areas than human tissue, but otherwise it matched much better with human histology with only minimal necrosis (Figure 1L).

Kaplan–Meier curves show the survival of the KPC and KPPC animals (Figure 1N,O). The median life expectancy of KPC mice was 28 weeks, which is significantly higher than that of KPPC mice (10 weeks; Figure 1N,O).

### 3.2. Pronounced Neural Remodeling in KPPC Animals

**Figure 2:**
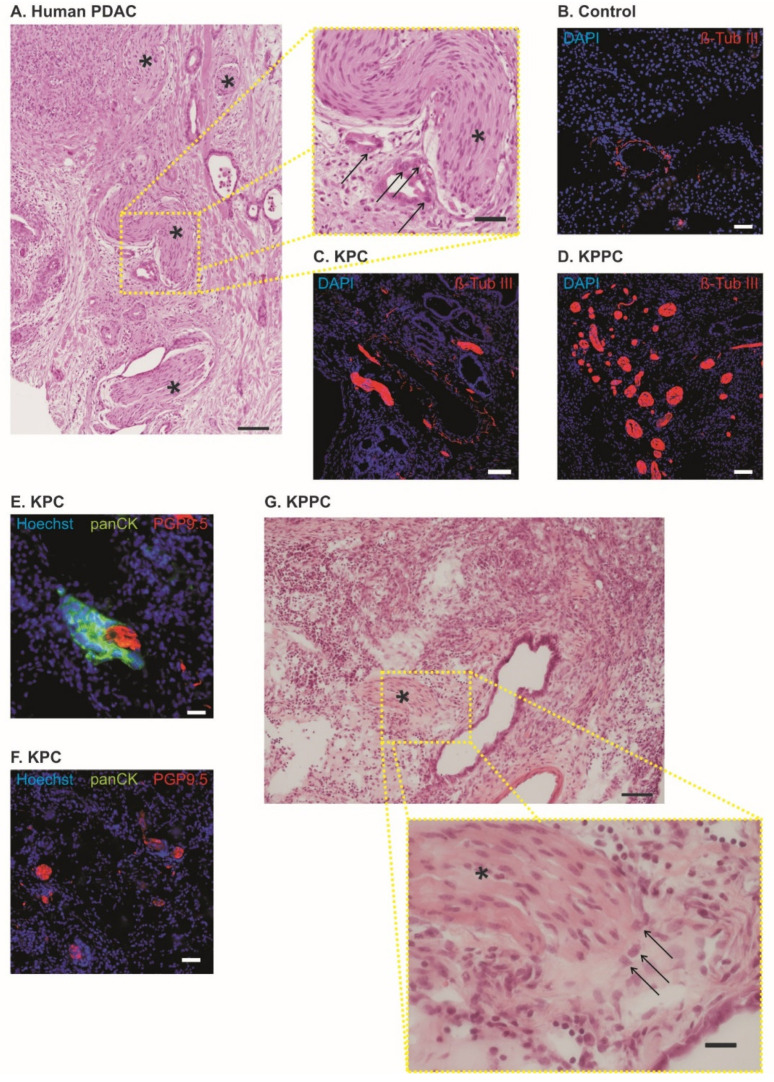
**KPC mice and KPPC mice show nerve fiber hypertrophy and increased fiber density**. (**A**). Human pancreatic cancer specimen with nerve fiber hypertrophy and increased nerve fiber density; Asterisk = Nerve; Arrow = PDAC (Scale bar = 100 µm in low magnification and 40 µm in high magnification). (**B**–**D**). Representative images of neuronal structures (Anti-beta-Tub III) in control (**B**), KPC mice (**B**) and KPPC mice ((**D**); scale bar = 100 µm). (**E**,**F**). Co-stating of PGP 9.5 (neuronal marker) with panCK (marker for PDAC) in tissue from KPC mice. (**G**), H&E staining in KPPC tissue showing cancer cells (arrows) invading the neuronal compartment (asterisk). Scale bar: 100 µm in low magnification and 10 µm in high magnification.

Similar to human tissue samples from PDAC patients (Figure 2A), KPC and KPPC mice showed significant nerve remodeling in the pancreas. In healthy pancreas, only few nerve fibers with small diameter could be identified (representative image in Figure 2B). In contrast, we could detect a huge nerve fiber hypertrophy and increased fiber density in KPC and KPPC animals (representative images in Figure 2C,D). Furthermore, we identified neural invasion in both mouse models, as shown in Figure 2E–G. However, in contrast with human tissue, neural invasion is seen less frequently. Several nerves remain unaffected (Figure 2F).

**Figure 3:**
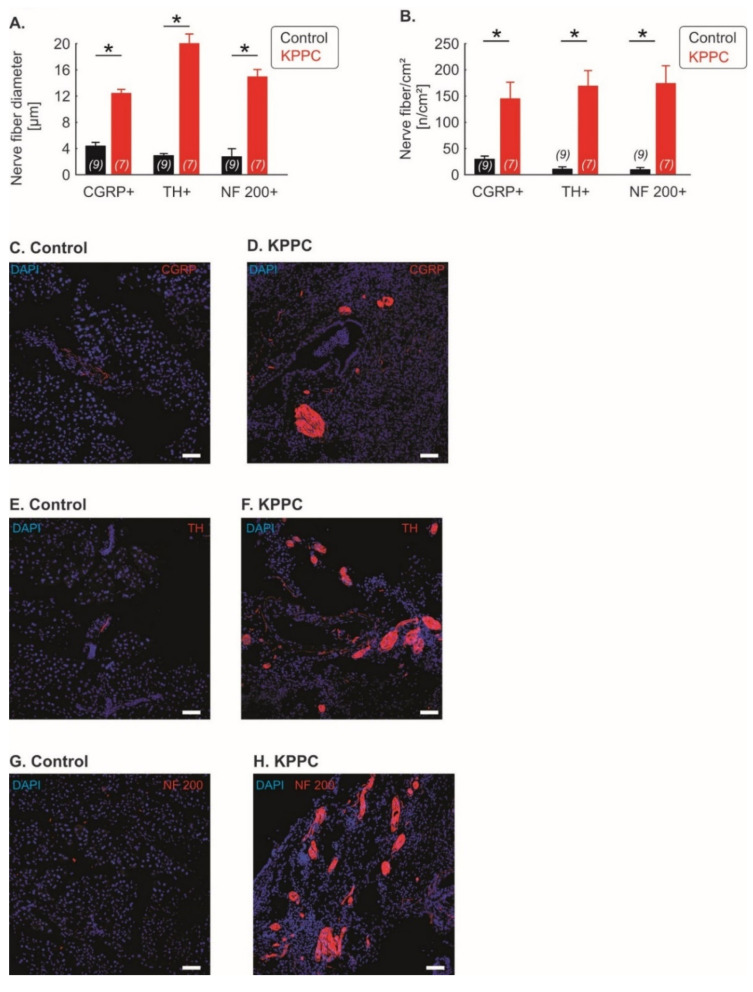
**Neuronal remodeling is detectable across several fiber classes**. (**A**–**H**): Quantification of the nerve fiber hypertrophy (**A**) and nerve fiber density (**B**) for Calcitonin Gene-Related Peptide (CGRP)-positive fibers (peptidergic nociceptive fibers), Tyrosine hydroxylase (TH)-positive fibers (sympathetic fibers and C-low threshold mechanoreceptors) and Neurofilament Protein 200 (NF200)-positive fibers (A beta fibers). The number of investigated mice is given in brackets. Representative images are shown in (**C**–**H**) (scale bar = 100 µm). Data are presented as mean ± S.E.M. * *p* < 0.05 in comparison between KPPC and control mice, Student’s *t*-test.

Subsequently, we investigated whether nerve remodeling is based on certain fiber types. We found a significant nerve fiber hypertrophy in fibers positive for Calcitonin Gene-Related Peptide (CGRP), which is a marker for peptidergic nociceptive fibers, as well as fibers positive for Tyrosine hydroxylase (TH), which labels sympathetic nerve fibers and a class of C fibers that function as low-threshold mechanoreceptors; interestingly, fibers positive for Neurofilament Protein 200 (NF200), which is strongly expressed in low threshold A fiber mechanoreceptors (Aß LTMRs), which sense non-noxious tactile inputs, also showed hypertrophy (each *p* < 0.001; Student’s *t*-test; Figure 3A). In addition, all three fiber types showed a significantly increased density of nerve fiber across pancreatic tissue (each *p* < 0.001; Student’s *t*-test; Figure 3B). Representative figures are shown in Figure 3C–H.

### 3.3. KPC, but Not KPPC Mice Show Signs of Abdominal Hypersensitivity

Using von Frey filaments, we evaluated if KPC or KPPC mice show signs of mechanical hypersensitivity at the abdomen similar to the clinical conditions.

**Figure 4:**
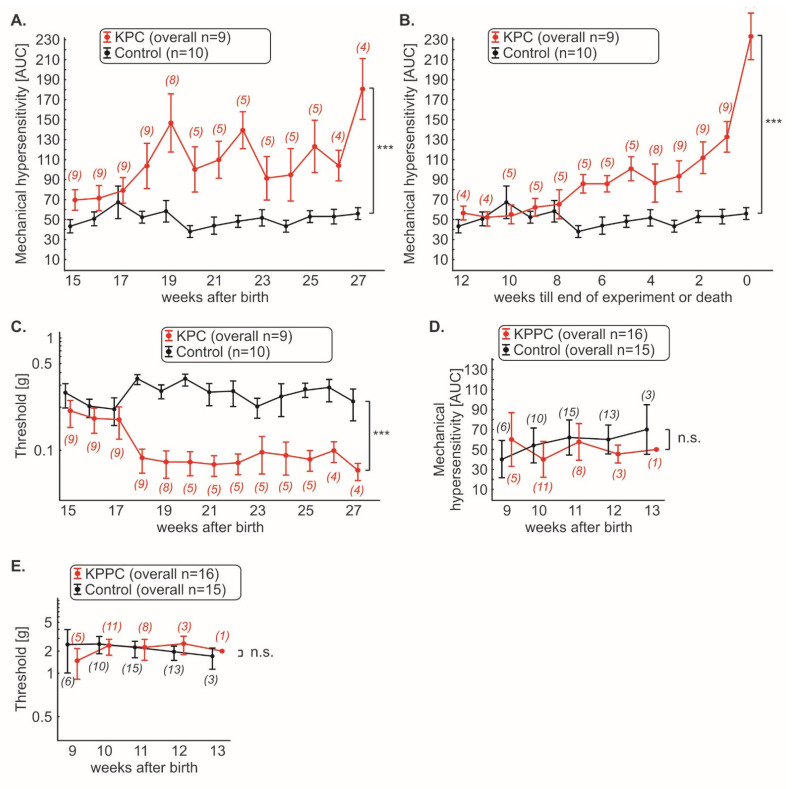
**KPC mice, but not KPPC mice, demonstrate abdominal hyperalgesia.** Mechanical hypersensitivity in KPC and KPPC mice was quantified weekly with von Frey filaments and shown as cumulative integral of responses to abdominal stimulation (AUC) to filament strengths of 0.008 g–0.16 g (**A**,**B**,**D**) or threshold (**C**,**E**). The number of mice used for the experiments varies over time and is given in brackets. Data are presented as mean ± S.E.M. Abbreviations: AUC: area under curve (see methods); n.s.: not significant. *** *p* < 0.001 in comparison between KPC or KPPC animals and control mice, ANOVA of repeated measures (**A**–**E**).

KPC mice showed increasing mechanical abdominal hypersensitivity with the course of disease compared to wildtype mice (Figure 4A; *p* < 0.05; ANOVA of repeated measures). However, the course of abdominal pain appeared to be discontinuous (Figure 4A). Therefore, we plotted the progression of tumor pain backward, starting with the last test performed (last test before death or planned end of experiment at week 27; Figure 4B). Thus, we noted continuously increasing tumor pain, which was clearly progressive, especially in the last weeks of the disease (Figure 4B; *p* < 0.05; ANOVA of repeated-measures). Furthermore, we found that cancer pain can be detected starting 18 weeks after birth (Figure 4A,C). At this time point, a rapid drop in mechanical thresholds was detectable (Figure 4C; *p* < 0.05; ANOVA of repeated-measures).

Surprisingly, in contrast, KPPC mice did not show signs of mechanical hypersensitivity (Figure 4D; *p* > 0.05; ANOVA of repeated measures). Furthermore, their pain thresholds were identical to those of control mice (Figure 4E; *p* > 0.05; ANOVA of repeated measures).

### 3.4. KPPC, but Not KPC Mice Show Signs of Cancer Associated Reduced Well-Being

**Figure 5:**
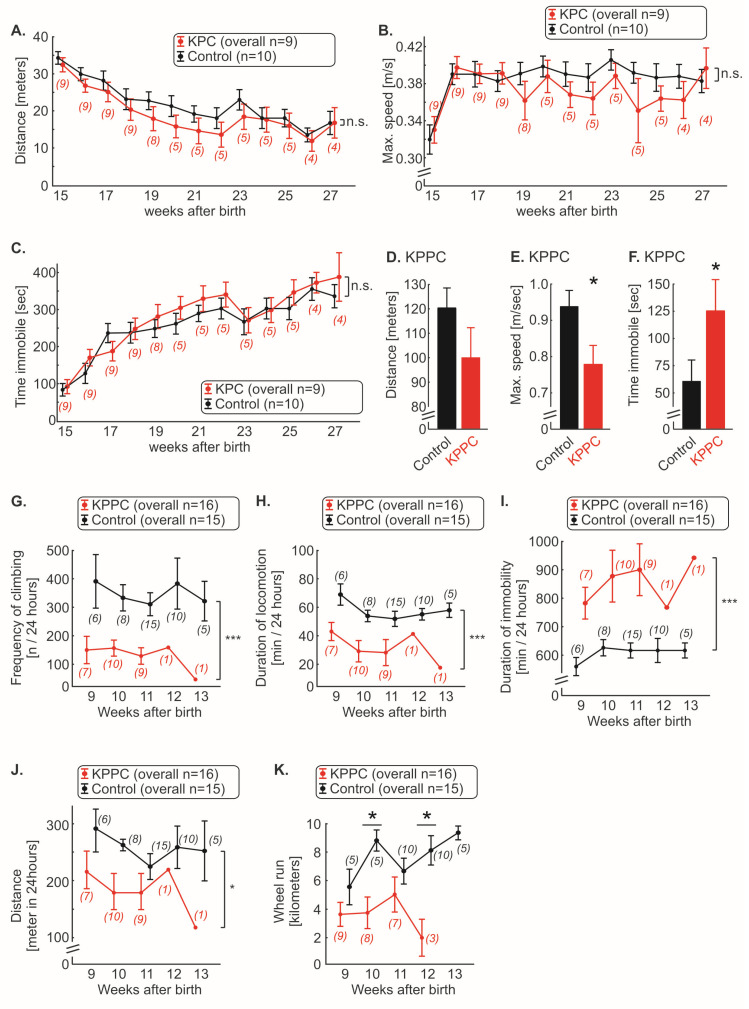
**KPPC mice, but not KPC mice, show reduced well-being.** (**A**–**F**). Behavioral analyses in the open field test. KPC mice (**A**–**C**) were tested weekly, and running distance in 10 min (**A**), maximum speed (**B**) and time immobile (**C**) was plotted. KPPC mice were tested at a single time point (9–10 weeks after birth) because they died soon after the start of the experiment (**D**–**F**). Running distance in 10 min (**D**), maximum speed (**E**) and time immobile (**F**) were determined (KPPC: n = 13; control: n = 15). (**G**–**J**). Automated quantification of frequency of climbing (**G**), duration of locomotion (**H**), duration of immobility (**I**) and total distance (**J**) were quantified weekly in KPPC mice with the LABORAS 24 h home cage monitoring system. (**K**). Weekly voluntary wheel running activity was recorded in KPPC mice and the absolute running distance within 24 h was determined. (**A**–**C**,**G**–**K**): The number of mice used at certain time points for the experiments varies over time and is given in brackets (see Figure 1N,O and Section 2). Data are presented as mean ± S.E.M. Abbreviations: n.s.: not significant. * *p* < 0.05 and *** *p* < 0.001 in comparison between KPC or KPPC with control mice, ANOVA of repeated measures (**A**–**C**,**G**–**J**) or Student’s *t*-test (**D**–**F**) or one-way ANOVA of random measures (**K**).

We therefore carried out experiments that reflect the changes in well-being of the animals. Using the open field test, we quantified the overall ambulatory activity. In KPC mice, we could not observe any significant changes in walking distance, running speed or time spent immobile over the entire course of disease (Figure 5A–C; n.s.; ANOVA of repeated measures). Since the KPPC mice died faster, they were analyzed in open field test at the single time point of 9–10 weeks after birth. KPPC mice showed a tendency to shorten their total walking distance (*p* > 0.05; Student’s *t*-test; Figure 5D) and a significant reduction in the maximum running speed, and spent more time immobile (*p* < 0.05; Student’s *t*-test; Figure 5E,F), which reflects reduced well-being in these animals.

In a home cage setting (Laboras), a significant reduction in climbing frequency and locomotion was observed in KPPC mice (each *p* < 0.05; ANOVA of repeated measures; Figure 5G,H). The duration of immobility was increased by approximately 50% in KPPC animals compared to wild-type mice (*p* < 0.05; ANOVA of repeated measures; Figure 5I). These three parameters (climbing, locomotion and immobility) were significantly different from the first test performed 9 weeks after birth till the last experiment (Figure 5G–I). Overall, a significant reduction in the total walking distance was observed (*p* < 0.05; ANOVA of repeated measures; Figure 5J).

Finally, in the voluntary wheel running test, KPPC mice ran shorter distances compared to control mice, in which we could measure significant reduction at 10 and 12 weeks after birth (each *p* < 0.05; one-way ANOVA of random measures; Figure 5K).

### 3.5. Several Neuroinflammatory Cytokines Are Overexpressed in KPPC Animals

**Figure 6:**
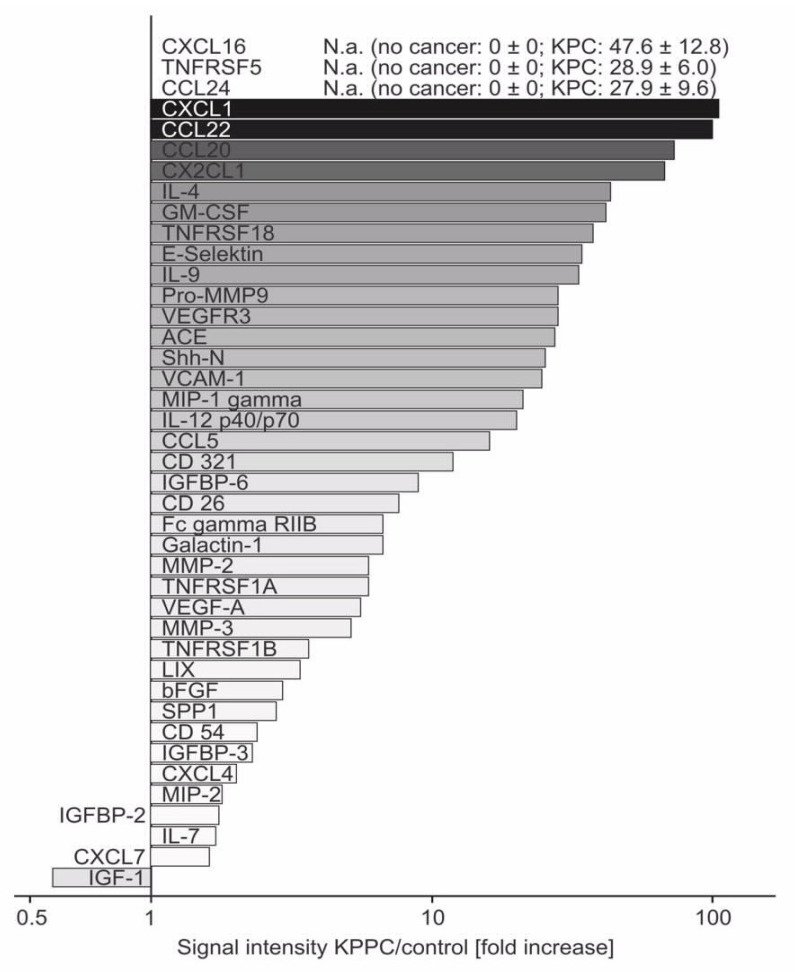
**Increased expression of several neuroinflammatory cytokines in pancreatic tissue of KPPC mice.** Fold changes of significantly altered cytokines upon analysis of expression of 144 mouse cytokines in freshly lysed pancreatic tissue of KPPC or control mice. The signal intensity measured for each cytokine was analyzed in control pancreas and in pancreatic tissue of KPPC mice. The fold-increased signal intensity in KPPC mice was calculated according to the formula Signal intensity_(KPPC)_/Signal intensity_(control)_ and presented in this figure. Abbreviations: N.a.: not available because Signal intensity_(control)_ = 0. Eight KPPC mice and six control mice were used. The original data (mean+/− S.E.M.) and the statistical values are given are given in Supplementary Table S1.

Because KPPC mice showed a pancreatic disease pathology that was closer to human PDAC and developed striking changes that reflected a state of overall malaise and low quality of life, we hypothesized the existence of neuroinflammatory changes. We therefore analyzed the expression of neuroinflammatory cytokines [28,29,30] using an array of 144 different cytokines in pancreatic tissues derived from KPPC and control animals. The detailed results with corresponding statistical analyses are shown in Supplementary Table S1. All cytokines that were subject to significant up- or down-regulation in KPPC mice compared to control mice are summarized in Figure 6 in the form of fold changes. Since CXCL16, CD40 and CCL24 were not detected in control tissue, no ratio can be given for these cytokines. Including these, the ten most upregulated cytokines comprise six chemokines, namely CXCL16, CCL24, CXCL1, CCL22, CLL20 and CX2CL1 (Figure 6 and Supplementary Table S1). In addition, two members of the tumor necrosis factor receptor super family (TNFRSF5 and TNFRSF18), IL-4 and GM-CSF showed marked over-expression in KPPC mice (Figure 6 and Supplementary Table S1).

## 4. Discussion

The aim of this study was to characterize genetic models of PDAC for their applicability in analyzing diverse pathophysiological aspects of the disease, in view of working out molecular and cellular underpinnings. In particular, these analyses are of great translational importance, as it is currently unknown whether genetically engineered mouse models can be used to study cancer pain and the profound changes in quality of life that come about in pancreatic cancer. The findings that merit note and discussion are: (I) The histological pattern in KPC mice and KPPC mice resembles that of the human condition, mimicking the duct-like structures of PDAC and showing hardly any necrotic areas, better than orthotopic mouse models. (II) Neuronal remodeling (e.g., hypertrophy and increased nerve fiber density) is pronounced in both genetically engineered mouse models and is fiber-class independent. (III) The rate and intensity of development of disease pathology has a major impact on manifestation of cancer-induced abdominal pain and reduced well-being, despite the similarity of the nature of the underlying pathology, as reflected by major differences between KPC mice and KPPC mice. (IV) Marked neuroinflammatory changes are evident in the tumor tissue.

Fortunately, our preclinical understanding of cancer pain in PDAC has increased rapidly in recent years [3,7,28,30,31,32]. It can be assumed that destruction of the neuronal compartment by cancer cells with neuronal remodeling is primarily responsible for this excruciating cancer pain [7]. Neuronal remodeling is characterized by hypertrophy, elongation and sprouting of nerve fibers, and strongly correlates with the development of cancer pain in PDAC [7,8]. As recently published data show, mediators released by neurons probably play an important first step in the attraction of cancer cells, but the interaction between cancer cells and nerves is not yet completely understood and is probably far more complex than our current knowledge reflects [28,30]. However, once cancer cells invade the neuronal structure, several adverse events take place. These include, in particular, neuronal inflammation, the release of neurotrophic growth factors and parenchymal immune cell infiltration, which may trigger neuronal remodeling [3,33,34]. To translate our findings into clinical studies and to better understand the complex interaction between pancreatic cancer cells and neurons, mouse models are needed to bridge the gap between in vitro experiments and patients.

To this end, we have characterized two genetically engineered mouse models which carry mutations in p53 and Kras. These mutations are not only used in many pancreatic cancer mouse models, but are also of great clinical relevance [2]. We found that the genetically engineered mouse models show significantly more similarities to human samples than mice with orthotopically injected K8484 cancer cells. Thus, we found characteristic duct-like structures and virtually no necrosis when tumor developed spontaneously and gradually, while necrosis formed in a time-dependent manner after injection of K8484 cells and is probably a sign of artificial tumor engraftment. One major implication of this finding is that packing of cells together in a small space likely leads to ischemic conditions, and thereby causes necrosis; in turn, tissue ischemia can elicit pain by activating or sensitizing nociceptors. Thus, pain in response to orthotopic injection of tumor cells may have mechanistic components that are not shared by the human cancer disorder. KPC mice and KPPC mice exhibited a significant amount of desmoplasia, which is a characteristic finding in human samples [2]. Furthermore, neuronal remodeling is an important characteristic of human PDAC tissue [8]. In the KPC and KPPC models, we could detect both hypertrophy and increased nerve fiber density, which is similar in magnitude to the findings in human PDAC. Overall, therefore, the genetic models reflect the course and nature of human disease pathology more accurately than the orthotopic model.

To the best of our knowledge, this is the first study that reports direct quantitative testing of cancer-induced abdominal hypersensitivity in the genetic models of PDAC. A previous study addressed exploratory behavior in the open field (Stopczynski et al. [35]), and reported changes in vertical motion, speculating that these might result from pain. Here, we report that KPC mice robustly show signs of abdominal cancer pain. The temporal onset of cancer pain may vary from animal to animal. However, when analyzed backwards, from the time of death or an advanced stage of tumor (in our experiment, 27 weeks after birth), there is an exponential increase in cancer pain with progression of the disease (Figure 3B). Thus, the findings in the animal model are strikingly similar to those reported by patients [3]. Many patients do not initially report pain. However, as the disease progresses, more and more patients develop cancer pain. In the final phase, up to 80% of patients suffer from cancer pain, which is often described as extreme [3]. We therefore consider the KPC mouse model to be very suitable for studying the onset and progression of cancer-associated pain in PDAC. The late onset of cancer pain, when PDAC tumors grow spontaneously and gradually in vivo, indicates that it is not a promising candidate as an early warning sign of readout for tumor growth.

The lack of signs of abdominal hyperalgesia in KPPC mice in our comprehensive behavioral analysis is indeed puzzling. Since these mice developed a marked reduction in well-being so quickly after tumor onset, it is possible that they were unable to exhibit nocifensive behaviors due to a general weakness. Alternatively, this striking phenotypic difference may result from yet-to-be-uncovered differences in molecular mediators and signaling in the complex cancer microenvironment when the tumors grow as a result of heterozygous or homozygous p53 mutations. For example, recent studies on other forms of cancer, such as melanomas, indicate that tumor cells can actively switch off activation and signaling in nociceptors in the tumor milieu via the checkpoint pathway [25,36]. In future studies, it will be interesting to perform screens probing molecular differences in the tumor milieu between KPC and KPPC models.

Our results do indicate a major value in employing the KPPC mouse model, however, since cancer-associated debilitating loss of quality of life was accurately modeled in these mice. Our results now demonstrate that malaise, reduced activity and reduction in well-being are not entirely abstract. They can be studied objectively and at least semi-quantitatively in the animal model, which is an important prerequisite for mechanistic analyses. As a start in this direction, we undertook screening for neuroinflammatory mediators, such as key cytokines, in the pancreatic milieu and observed increased levels of several known mediators of tumor–nerve interactions. This topic is increasingly coming into scientific focus for several reasons. On the one hand, nerve fibers serve as a route for ultra-early micrometastasis (NEX phenomenon) and at the same time serve as a protected compartment for the tumor with a reduced immune response [34,37]. Moreover, after tumor cells grow into the neuronal compartment, there is gradual destruction of it and neuronal remodeling develops [7,8]. Attraction of tumor cells represents the first step in neuronal invasion. Chemokines released by neurons seem to play an important role in this process [28,30]. We also observed a significant overexpression of several chemokines, e.g., CX2CL1 and CXCL16. The relevance of the identified cytokines is shown, for example, by CX2CL1 (67-fold upregulation in KPPC tissue), which is already known to play a relevant role in cancer–nerve interaction. Marchesi et al. demonstrated that CX2CL1 is released by neurons and that cancer cells which overexpress the corresponding receptor invade the neuronal compartment. High expression of the CX2CL1 receptor on tumor cells was associated with early tumor recurrence [31]. In addition, CX2CL1 has an important impact on the development of neuropathic pain, which is often observed in patients with PDAC [38,39]. In patients with breast cancer, cancer-associated fibroblasts have recently been shown to induce attraction and migration of cancer cells through secretion of CXCL16 [40]. In other tumor entities, CXCL16 plays a relevant role in the interaction between tumor cells and the microenvironment. In colon carcinoma, for example, it has been shown that colorectal tumor cells manipulate healthy neighboring cells via CXCL16 in exosomes, and can thus increasingly metastasize [41]. Accordingly, the serum level of CXCL16 correlates with metachronous liver metastasis and poor prognosis [42]. Targeting chemokines or chemokine receptors in tumor diseases is an innovative therapeutic strategy, and the first clinical trials report promising outcomes [43]. Furthermore, we identified several other cytokines that are also massively upregulated and whose role in the interaction between tumor cells and the microenvironment is still largely unknown. They represent interesting new mediators whose investigation will contribute significantly to a better understanding of the disease.

Finally, we would like to discuss a limitation of the study. p53 and Kras mutations are the most common mutations in human tissue [13]. Therefore, we had chosen these two mutations. However, a variety of other mutations exist, especially in tumor suppressor genes, such as CDKN2A, SMAD4, ARID1 or BRCA2 [13]. Whether mutations in these genes develop an even more distinct phenotype is still unknown and should be further investigated. In summary, we found that KPC and KPPC mouse models have striking similarities to human PDAC. They develop neuronal remodeling and can be used to study cancer pain (KPC) and quality of life parameters (KPPC), rendering them highly suitable for behavioral and mechanistic analyses of these diverse facets of cancer and its consequences. Several cytokines were found to be elevated in tumor tissue, which may play a role in the cancer–nerve interactions.

## Supplementary Materials

The following are available online at https://www.mdpi.com/article/10.3390/cells11172634/s1, Table S1: Increased expression of several cytokines in pancreatic tissue of KPPC mice.
