# Supplementary material for: Genetic Mouse Models to Study Pancreatic Cancer-Induced Pain and Reduction in Well-Being
Source: Cells. 2022 Aug 24;11(17):2634. doi: 10.3390/cells11172634 (PMC9454877; doi:10.3390/cells11172634)
Supplement: Supplementary file 1 [file cells-11-02634-s001.zip › cells-1852905-supplementary.pdf]

SUPPLEMENTARY MATERIAL

Suppl. Table 1: Increased expression of several cytokines in pancreatic tissue of KPPC mice

Post mortem we isolated the pancreatic gland including the pancreatic carcinoma and lysed it immediately. After normalizing to the total protein amount, we performed a broad semi-quantitative mouse cytokine array (RayBio® C-Series Mouse Cytokine Antibody Array C2000; #AAM-CYT-2000) according to the manufacturer's instructions. Duplicates of each cytokine were analyzed in eight KPPC mice and six control mice ("Control"). The signal intensity measured for each cytokine was analyzed in control pancreas ("Control") and in pancreatic tissue of KPPC mice. The signal intensity of each dot was normalized to the positive control (= 100).

Data are presented as mean ± SEM. The increase in KPPC tissue was calculated according to the formula Signal $\text{intensity}_{(KPPC)}$ / Signal $\text{intensity}_{(control)}$. Abbreviations: NA: not available because Signal $\text{intensity}_{(control)}$ = 0. * or ** or ***: P< 0.05 or < 0.01 or < 0.001 in comparison between Signal $\text{intensity}_{(KPPC)}$ and Signal $\text{intensity}_{(control)}$; analyzed via Mann-Whitney-*U*-test.

Table S1: Increased expression of several cytokines in pancreatic tissue of KPPC mice 1.

Antigen	Signal intensity **Control**	Signal intensity **KPPC**	Signal intensity$_{(KPPC)}$ / Signal intensity$_{(control)}$
CXCL16	0 ± 0	47.6 ± 12.8	N.a.***
TNFRSF5 (CD40)	0 ± 0	28.9 ± 6.0	N.a.***
Eotaxin-2 (CCL24)	0 ± 0	27.9 ± 9.6	N.a.*
KC (CXCL1)	0.3 ± 0.3	32.6 ± 5.9	104***
MDC (CCL22)	0.5 ± 0.5	49.6 ± 4.7	98***
MIP-3 alpha	0.2 ± 0.1	16.6 ± 3.2	72***
Fractalkine	0.2 ± 0.1	16.3 ± 5.0	67*
IL-4	1.0 ± 0.6	41.2 ± 7.8	43**
GM-CSF	0.2 ± 0.1	10.1 ± 1.5	41**
GITR (TNFRSF18)	0.2 ± 0.1	9.2 ± 2.6	37**
E-Selektin	0.2 ± 0.1	8.1 ± 1.0	34***
IL-9	0.2 ± 0.1	8.0 ± 2.2	33**
Pro-MMP9	0.2 ± 0.1	6.9 ± 1.2	28***
VEGFR3	0.3 ± 0.1	7.3 ± 1.5	28**
ACE	2.5 ± 1.1	69.1 ± 8.9	27**
Shh-N	0.4 ± 0.3	9.4 ± 3.0	25*
VCAM-1 (CD106)	5.3 ± 2.8	128.0 ± 17.9	24***
MIP-1 gamma	19.7 ± 4.0	414.6 ±70.3	21***
IL-12 p40/p70	0.3 ± 0.1	5.7 ± 0.8	20**
RANTES (CCL5)	0.3 ± 0.1	4.7 ± 1.3	16**
JAM-A (CD321)	4.3 ± 0.3	51.7 ± 13.7	12*
IGFBP-6	1.7 ± 1.1	15.5 ± 3.6	8.9*
CD26	0.9 ± 0.6	6.8 ± 1.7	7.6*
Fc gamma RIIB	12.3 ± 7.0	82.9 ± 14.1	6.7***
Galactin-1	16.4 ± 1.8	109.1 ± 5.6	6.6*
MMP-2	11.0 ± 3.6	65.6 ± 4.3	6.0***
TNF RI	2.5 ± 2.5	14.8 ± 3.1	5.9**
VEGF-A	1.1 ± 0.9	6.3 ± 1.4	5.6*
MMP-3	2.1 ± 1.1	11.0 ± 1.3	5.1***
TNF RII	10.2 ± 4.7	37.4 ± 3.5	3.6***
LIX	18.5 ± 7.5	62.9 ± 12.1	3.4**
bFGF	36.6 ± 11.0	107.9 ± 8.6	2.9***
OPN (SPP1)	52.8 ± 6.8	146.6 ± 13.4	2.8***
ICAM-1 (CD54)	43.0 ± 6.2	102.6 ± 6.4	2.4***
IGFBP-3	30.0 ± 14.2	68.5 ± 10.7	2.3*
PF-4 (CXCL4)	28.0 ± 8.6	56.4 ± 4.6	2.0*
MIP-2	38.3 ± 11.6	68.3 ± 7.9	1.8*
IGFBP-2	29.1 ± 4.7	50.9 ± 4.5	1.7**
IL-7	5.9 ± 0.6	10.0 ± 0.7	1.7**
TCK-1 (CXCL7)	66.2 ± 13.4	107.2 ± 9.9	1.6*
IGF-1	97.5 ± 16.0	58.4 ± 9.2	0.6*

Table S1 continued

Antigen	Signal intensity **Control**	Signal intensity **KPPC**	Signal intensity(KPPC) / Signal intensity(control)
Axl	0.2 ± 0.1	9.1 ± 3.3	45 n.s.
BLC (CXCL13)	0.1 ± 0.1	5.8 ± 2.7	48 n.s.
CD30 Ligand	0 ± 0	1.3 ± 1.3	N.a. n.s.
CD30 (TNFSF8)	0 ± 0	0 ± 0	N.a. n.s.
CRG-2	0 ± 0	2.1 ± 2.2	N.a. n.s.
CTACK (CCL27)	38.0 ± 11.8	30.8 ± 5.4	0.8 n.s.
Eotaxin-1 (CCL11)	3.1 ± 1.8	5.0 ± 4.2	1.7 n.s.
Fas Ligand (TNFSF6)	0 ± 0	1.9 ± 0.9	N.a. n.s.
GCSF	0 ± 0	3.7 ± 3.4	N.a. n.s.
IFN-gamma	0 ± 0	0.4 ± 0.4	N.a. n.s.
IGFBP-5	17.1 ± 9.1	9.8 ± 2.1	0.5 n.s.
IL-1 alpha (IL-1 F1)	20.1 ± 10.6	23.9 ± 3.8	1.1 n.s.
IL-1 beta (IL-1 F2)	0 ± 0	0 ± 0	N.a. n.s.
IL-2	0 ± 0	0 ± 0	N.a. n.s.
IL-3	0 ± 0	0 ± 0	N.a. n.s.
Il-3 R beta	0.5 ± 0.4	0 ± 0	0 n.s.
IL-5	0 ± 0	2.3 ± 1.5	N.a. n.s.
IL-6	0 ± 0	3.8 ± 2.5	N.a. n.s.
IL-10	0 ± 0	0 ± 0	N.a. n.s.
IL-12 p70	21.6 ± 11.0	36.6 ± 4.9	1.7 n.s.
IL-13	4.4 ± 2.5	10.2 ± 2.1	2.2 n.s.
IL-17A	0 ± 0	2.4 ± 1.7	N.a. n.s.
Leptin R	0 ± 0	3.7 ± 1.3	N.a. n.s.
Leptin	17.2 ± 8.6	12.6 ± 2.6	0.7 n.s.
L-Selectin (CD62L)	0 ± 0	3.4 ± 1.7	N.a. n.s.
Ltn (XCL1)	7.8 ± 4.0	18.7 ± 4.4	2.4 n.s.
MCP-1 (CCL2)	40.3 ± 13.2	55.7 ± 6.7	1.4 n.s.
MCP-5	8.8 ± 0.6	17.4 ± 2.9	2.0 n.s.
M-CSF	47.3 ± 17.1	48.9 ± 5.0	1.0 n.s.
MIG (CXCL9)	6.8 ± 4.1	8.4 ± 1.7	1.2 n.s.
MIP-1 alpha (CCL3)	0 ± 0	0.2 ± 0.1	N.a. n.s.
MIP-3 beta (CCL19)	2.8 ± 2.5	8.1 ± 1.7	2.9 n.s.
P-Selectin	24.3 ± 7.2	34.3 ± 3.3	1.4 n.s.
SCF	7.9 ± 2.7	6.9 ± 2.4	0.8 n.s.
SDF-1 alpha	21.1 ± 4.0	32.5 ± 6.3	1.5 n.s.
TARC (CCL17)	17.6 ± 17.6	21.3 ± 4.9	1.2 n.s.
I-309 (TCA-3/CCL1)	59.1 ± 20.5	64.8 ± 7.0	1.1 n.s.
TECK (CCL25)	0 ± 0	2.6 ± 1.0	N.a. n.s.
TIMP-1	30.0 ± 18.2	29.3 ± 3.3	1.0 n.s.
TNF alpha	2.6 ± 2.6	2.6 ± 2.1	1.0 n.s.
TPO	4.0 ± 2.0	10.2 ± 2.0	2.5 n.s.
Dtk	11.0 ± 2.7	16.1 ± 1.9	1.5 n.s.
Flt-3 Ligand	28.4 ± 11.6	11.0 ± 1.6	0.4 n.s.
HGFR	1.3 ± 1.2	2.1 ± 0.7	1.6 n.s.
IGF-2	4.9 ± 1.5	6.6 ± 0.6	1.3 n.s.
IL-15	1.1 ± 0.7	4.4 ± 1.4	3.7 n.s.
IL-17 RB	0.8 ± 0.5	1.5 ± 1.5	1.7 n.s.
I-TAC (CXCL11)	20.3 ± 4.6	22.9 ± 2.1	1.1 n.s.
Lungkine (CXCL15)	30.0 ± 6.4	38.8 ± 3.0	1.3 n.s.
OPG (TNFRSF11B)	1.2 ± 0.6	11.1 ± 4.8	9.2 n.s.
Resistin	17.8 ± 2.7	16.9 ± 1.5	0.9 n.s.

Table S1 continued

Antigen	Signal intensity **Control**	Signal intensity **KPPC**	Signal intensity(KPPC) / Signal intensity(control)
TIMP-2	11.5 ± 2.2	15.3 ± 1.6	1.3 n.s.
TRANCE (TNFSF11)	15.1 ± 3.4	18.9 ± 1.7	1.3 n.s.
TROY (TNFRSF19)	27.2 ± 9.0	26.3 ± 1.9	1.0 n.s.
TSLP	0 ± 0	0 ± 0	N.a. n.s.
VEGFR1	41.2 ± 15.6	40.0 ± 4.2	1.0 n.s.
VEGFR2	0 ± 0	3.0 ± 0.9	N.a. n.s.
VEGF-D	0 ± 0	1.3 ± 0.9	N.a. n.s.
4-1BB (TNFRSF9)	0 ± 0	1.8 ± 1.2	N.a. n.s.
6Ckine (CCL21)	0.1 ± 0.1	6.6 ± 6.4	48.9 n.s.
ALK-1	3.4 ± 0.5	19.3 ± 6.0	5.7 n.s.
AR	5.3 ± 1.9	21.1 ± 2.4	4.0 n.s.
CT-1	2.7 ± 2.6	3.7 ± 2.8	1.4 n.s.
CD27 (TNFRSF7)	0 ± 0	8.4 ± 6.3	N.a. n.s.
CD27 ligand	0 ± 0	0 ± 0	N.a. n.s.
CD36 (SR-B3)	6.5 ± 2.5	36.2 ± 9.6	5.5 n.s.
CD40 ligand	9.4 ± 2.0	41.8 ± 10.1	4.4 n.s.
Chordin	0 ± 0	0 ± 0	N.a. n.s.
CTLA-4 (CD152)	1.6 ± 0.8	24.6 ± 8.9	15.1 n.s.
Decorin	44.8 ± 26.5	92.9 ± 5.9	2.1 n.s.
DKK-1	2.6 ± 1.3	21.4 ± 5.1	8.2 n.s.
E-Cadherin	19.5 ± 14.7	100.8 ± 13.5	5.1 n.s.
EGF	0.9 ± 0.9	13.7 ± 11.9	15.1 n.s.
Endoglin (CD105)	1.1 ± 1.1	32.9 ± 8.8	29.3 n.s.
Epigen	0 ± 0	1.1 ± 1.1	N.a. n.s.
Epiregulin	3.5 ± 0.4	23.1 ± 9.7	6.5 n.s.
Gas1	3.5 ± 0.8	16.1 ± 4.3	4.6 n.s.
Gas6	7.0 ± 0.8	75.7 ± 13.1	10.8 n.s.
GITR ligand	6.3 ± 0.3	22.1 ± 8.1	3.5 n.s.
Granzyme B	5.0 ± 0.1	56.6 ± 13.8	11.3 n.s.
HAI-1	5.0 ± 1.8	23.4 ± 5.3	4.6 n.s.
HGF	0 ± 0	0 ± 0	N.a. n.s.
IL-1R4 (ST2)	34.0 ± 2.5	35.6 ± 9.8	1.0 n.s.
IL-11	29.1 ± 7.2	33.6 ± 12.7	1.1 n.s.
IL-17B	25.0 ± 11.6	39.4 ± 11.9	1.6 n.s.
IL-17E (IL-25)	8.0 ± 2.5	11.6 ± 6.7	1.4 n.s.
IL-17F	16.4 ± 8.2	18.4 ± 10.7	1.1 n.s.
IL-1 RA (IL-1 F3)	4.1 ± 1.6	138.8 ± 17.6	33.4 n.s.
IL-2 R alpha	4.3 ± 0.3	28.5 ± 16.5	6.5 n.s.
IL-20	0 ± 0	0.6 ± 0.6	N.a. n.s.
IL-21	6.1 ± 0.2	20.2 ± 5.0	3.2 n.s.
IL-28A	6.2 ± 0.1	34.6 ± 6.6	5.6 n.s.
IL-6R	4.2 ± 0.2	29.9 ± 7.3	7.1 n.s.
Mad-CAM-1	0 ± 0	0 ± 0	N.a. n.s.
MFG-E8	0 ± 0	0 ± 0	N.a. n.s.
Neprilysin	2.8 ± 1.2	2.2 ± 2.2	0.8 n.s.
Pentraxin-3 (TSG-14)	8.1 ± 4.1	5.9 ± 3.5	0.7 n.s.
Prolactin	12.0 ± 4.5	5.7 ± 3.6	0.5 n.s.
RAGE	0 ± 0	0 ± 0	N.a. n.s.
TACI (TNFRSF13B)	2.0 ± 1.5	4.5 ± 4.2	2.2 n.s.
TREM-1	25.8 ± 8.1	33.0 ± 5.9	1.3 n.s.
TWEAK (TNFSF12)	4.1 ± 1.2	5.8 ± 3.6	1.4 n.s.
TWEAK R	18.3 ± 6.2	29.4 ± 9.3	1.6 n.s.
